# Maturation of Cardiac Energy Metabolism During Perinatal Development

**DOI:** 10.3389/fphys.2018.00959

**Published:** 2018-07-19

**Authors:** Jérôme Piquereau, Renée Ventura-Clapier

**Affiliations:** Signalling and Cardiovascular Pathophysiology – UMR-S 1180, Université Paris-Sud, Institut National de la Santé et de la Recherche Médicale, Université Paris-Saclay, Châtenay-Malabry, France

**Keywords:** heart, mitochondria, creatine kinase shuttle, cytoarchitecture, substrates, fatty acids, postnatal, fetus

## Abstract

As one of the highest energy consumer organ in mammals, the heart has to be provided with a high amount of energy as soon as its first beats *in utero*. During the development of this organ, energy is produced within the cardiac muscle cell depending on substrate availability, oxygen pressure and cardiac workload that drastically change at birth. Thus, energy metabolism relying essentially on carbohydrates in fetal heart is very different from the adult one and birth is the trigger of a profound maturation which ensures the transition to a highly oxidative metabolism depending on lipid utilization. To face the substantial increase in cardiac workload resulting from the growth of the organism during the postnatal period, the heart not only develops its capacity for energy production but also undergoes a hypertrophic growth to adapt its contractile capacity to its new function. This leads to a profound cytoarchitectural remodeling of the cardiomyocyte which becomes a highly compartmentalized structure. As a consequence, within the mature cardiac muscle, energy transfer between energy producing and consuming compartments requires organized energy transfer systems that are established in the early postnatal life. This review aims at describing the major rearrangements of energy metabolism during the perinatal development.

## Introduction

The emergence, in the course of evolution, of individuals whose existence is based on the coordinated functioning of different organs is fascinating. This complex organization is established during organogenesis which is the scene of cell differentiations and specializations leading to the creation of organs with specific functions. These entities, immature at the end of organogenesis, undergo adaptations to the needs of the organism in its environment during prenatal and postnatal life maturation. The heart, which ensures proper distribution of oxygen and nutrient according to the needs of the body throughout life, also undergoes essential maturation processes. These are obvious in the prenatal period during which the primitive tubular heart matures to a four-cavity organ (Brand, 2003; Sedmera and McQuinn, 2008). This morphological adaptation mainly results from a massive proliferation of cardiac muscle cells that relies on coordinated biosynthetic pathways (Christoffels et al., 2000; Lunt and Vander Heiden, 2011).

During the postnatal period, numerous structural and metabolic modifications of the cardiac muscle cell allow adaptation to the new requirements of the body. In particular, while fetal life is characterized by the parallel operation of the two ventricles because of the strong resistance of the collapsed lungs, the sudden expansion of the pulmonary alveoli at birth induces major hemodynamic changes leading, in the early hours of *ex utero* life, to the series operation of the two ventricles (Heymann and Rudolph, 1975; Hew and Keller, 2003). This reorganization of the cardiovascular system in its new environment leads to changes in oxygen levels, pressure, charge and volume of the two ventricles. Consequently, essential maturation of the cardiomyocyte is necessary for optimization of the cardiac function.

In addition to the new circulation pattern, because of the rapid growth of the body, the postnatal period is characterized by a constant increase in cardiac workload. In response, the cardiac mass increases by hyperplasia (cell proliferation) then by hypertrophy (increase in the volume of each cardiomyocyte) (Clubb and Bishop, 1984; Hoerter et al., 1991; Li et al., 1996; Leu et al., 2001), however, not in proportion to body mass so that heart weight to body weight decreases during postnatal development (Hoerter et al., 1991). These changes require an increase in cardiac efficiency. This will be achieved by a profound complexification of cardiomyocyte architecture, excitation–contraction coupling and energy metabolism. This prompts to the establishment of more efficient energy production systems (Lopaschuk and Jaswal, 2010; Piquereau et al., 2010).

There is a strong link between myocardial metabolism and cardiac function since the heart is among the largest energy consumer organs in the body. Energy can be produced either from anaerobic glycolysis in the cytosol or from oxidative metabolism in mitochondria. Energy is stored in the form of adenosine triphosphate (ATP) and phosphocreatine (PCr) which is formed by the phosphorylation of creatine (Cr) from ATP by the creatine kinase (CK) reaction. The adult heart is the most efficient energy consuming organ with around 1 mM ATP consumed per second that should be produced on a “pay as you go” manner, as energy reserves in the form of ATP and PCr account only for a few seconds of activity. This energy is essentially provided by oxidative phosphorylations taking place in mitochondria (>90% for the adult heart). This is evidenced by the linear correlation between oxygen consumption and cardiac work (Stepanov et al., 1997).

The mature heart is omnivorous as it can metabolize carbohydrates, lipids, proteins, and lactate to produce energy. Lipids and carbohydrate deriving substrates produce reducing equivalents that are oxidized along the respiratory chain providing a proton gradient that is used as a driving force to produce ATP from ADP (Mitchell, 1979; Gautheron, 1984; Noji et al., 1997). The high lipid concentration in the blood and the high oxygenation play a predominant role in determining the metabolism of the adult heart (Fisher et al., 1980; Stanley et al., 2005; Lopaschuk et al., 2010). It thus mainly consumes lipids, and carbohydrates provide only 10–40% of mitochondria-oxidized acetyl-CoA (Stanley et al., 2005). The high mitochondrial mass and the optimal activity of the Krebs cycle and the respiratory chain enzymes ensure optimal conditions for energy production from fatty acids (Glatz and Veerkamp, 1982; Werner et al., 1982; Minai et al., 2008). This is reinforced by the complex reciprocal inhibition between lipid oxidation and carbohydrate oxidation known as the Randle cycle and involving allosteric control, reversible phosphorylation, and the expression of key enzymes (Hue and Taegtmeyer, 2009). The lipid metabolism displays a higher yield (in terms of energy production) but utilizes more oxygen per ATP produced than the glycolytic metabolism which is rather effective when glucose is supplied in abundance (Lunt and Vander Heiden, 2011). During development the metabolic profile of the heart evolves, taking advantage of the specificities of each metabolic orientation to face the specific conditions of *in utero* or *ex utero* life.

## How Different Is Energy Metabolism Between the Newborn and the Adult Heart?

While lipids and carbohydrates are the main sources of energy for cardiomyocytes (Williamson et al., 1976; Saks et al., 2006b; Ventura-Clapier et al., 2010), their respective utilization varies greatly during organ development (Lopaschuk et al., 1991; Makinde et al., 1998). At a given age, the metabolic orientation of the heart depends on one hand on the activity of enzymes involved in the energy pathways, but is also strongly influenced by the availability of the circulating substrates, themselves mainly dependent on nutrition. Thus, the energy demand, the oxygen content and the availability of substrates, among others, determine the orientation of cardiac metabolism.

**FIGURE 1:**
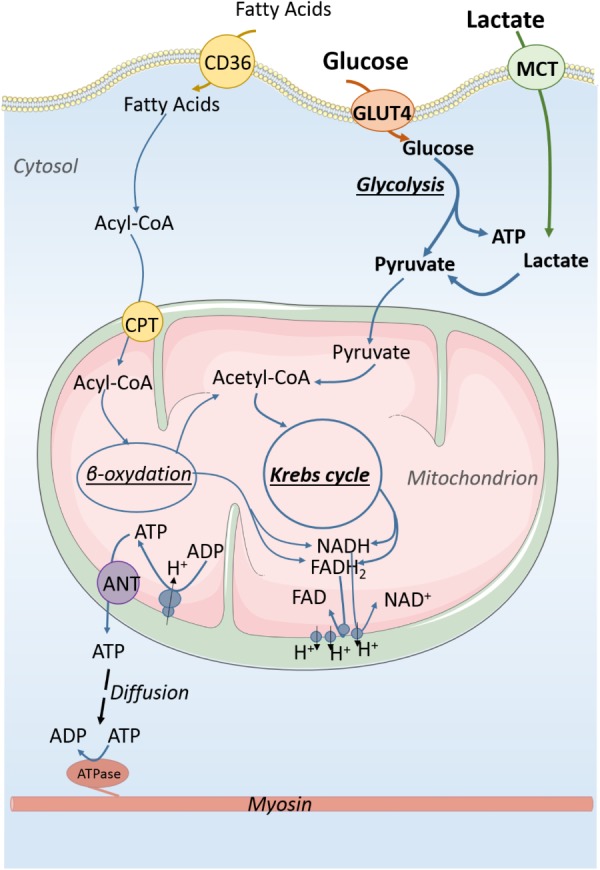
Energy production in immature cardiomyocyte. Carbohydrates are the main substrates for energy production of the immature cardiomyocyte. About 50% of ATP production derives from anaerobic glycolysis which also produces pyruvate that can be used by mitochondria to generate energy from oxidative phosphorylations. Lactate that is in high concentration in the fetal blood participates in energy production through its conversion to pyruvate. Energy is essentially produced in the form of ATP molecules which diffuse within the cytosol to reach ATP consumers of the cell. CD36, fatty acid translocase; GLUT4, glucose transporter 4; MCT, monocarboxylate transporter; ATP, adenosine triphosphate; ADP, adenosine diphosphate; NAD and NADH, oxidized and reduced nicotinamide adenine dinucleotide; FAD and FADH2, oxidized and reduced, flavin adenine dinucleotide. Acyl-CoA, acyl-coenzyme A.

**FIGURE 2:**
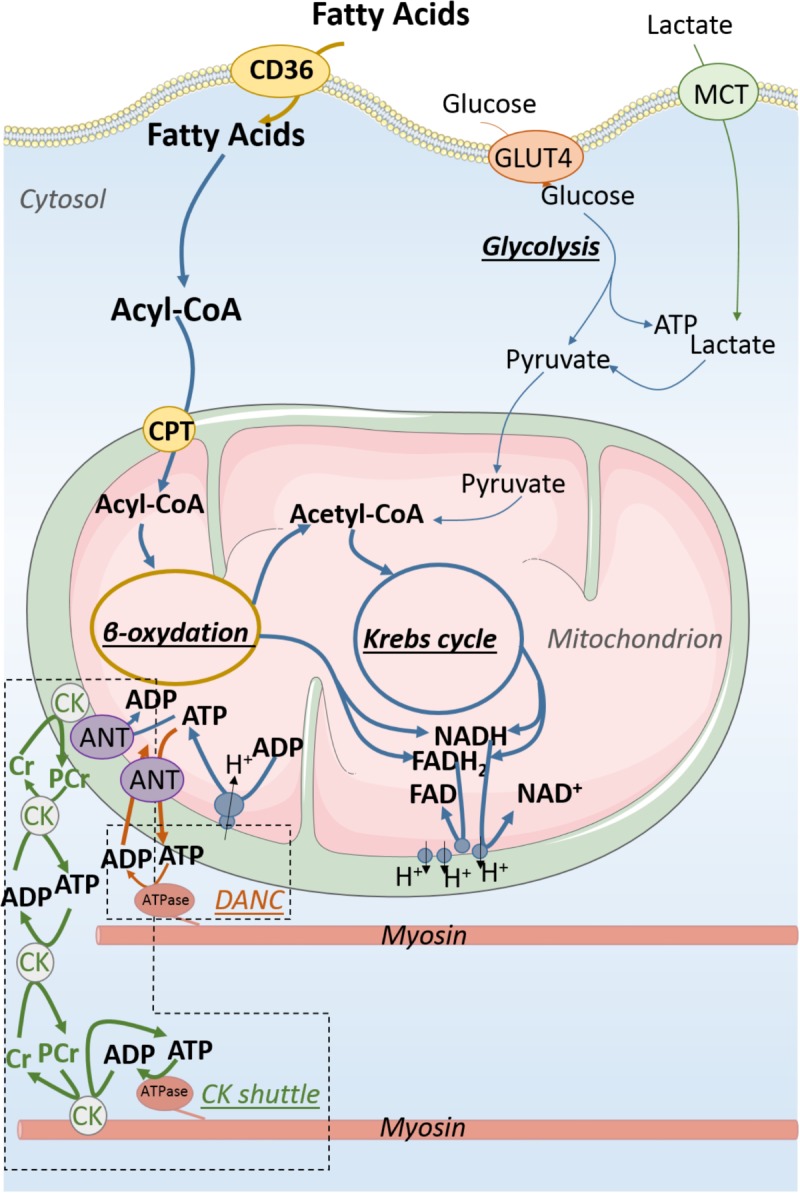
Energy production and transfer in adult cardiomyocyte. Fatty acids are far and away the main substrates for energy production of the adult cardiomyocyte when the relative contribution of carbohydrate is much smaller than in the fetal cardiomyocyte. About 90% of energy is provided by oxidative phosphorylations taking place in mitochondria. The major part of ATP produced by mitochondria is transformed into phosphocreatine (PCr) by mitochondrial creatine kinase (CK) which initiates energy transfer to energy consumers using the CK shuttle to overcome diffusion issue resulting from the packed intracellular organization of the adult cardiomyocyte. This complex intracellular architecture allows the emergence of energy micro-domains in which adenine nucleotides are directly exchanged between mitochondria and energy consumers (DANC).

The heart progresses from a high glycolytic activity in the early phases of development to an almost exclusive oxidative metabolism at maturity (Ascuitto and Ross-Ascuitto, 1996; Bartelds et al., 2000; Lopaschuk and Jaswal, 2010) (**Figures 1**,**2**). The low levels of circulating fatty acid and the high levels of lactate *in utero* (Portman et al., 1969; Girard et al., 1992; Bartelds et al., 1998; Makinde et al., 1998) contribute to establish the high glycolytic activity encountered in the fetal heart. Indeed, substrates play an essential role in metabolism since lactate hinders the oxidation of lipids while fatty acids are able to repress the processes involved in the use of carbohydrates (Werner et al., 1989). As a result in the fetus, only 15% of total energy production derives from the use of fatty acids (Lopaschuk and Jaswal, 2010), a substrate choice adapted to the low oxygen environment similar to the one encountered by the hypoxic adult hearts (Patterson and Zhang, 2010; Murray et al., 2014). In addition to the impact of the substrate availability on fetal metabolism, the higher activity and specific regulations of glycolytic enzymes in this stage of development (Jones and Rolph, 1985) favor the production of anaerobic ATP (Bristow et al., 1987; Ascuitto and Ross-Ascuitto, 1996). For example, the intracellular distribution of the hexokinase isoform (a glycolysis enzyme) expressed at birth is different from that of the adult one, contributing to the orientation of metabolism toward glycolysis (Calmettes et al., 2013).

In the fetal heart, thus 50% of ATP production derives from anaerobic glycolysis (Lopaschuk et al., 1991) the other part coming from oxidative phosphorylations. Although in the early stages of development, activity of the respiratory chain and mitochondrial mass are definitely lower (Werner et al., 1982; Minai et al., 2008), the fetal myocardium remains nonetheless endowed with real oxidative capacities (Warshaw, 1969; Muhlfeld et al., 2005). Indeed, early in cell differentiation, oxidative metabolism is necessary for establishing a functional cardiac phenotype in stem cells (Chung et al., 2007), showing the importance of energy metabolism for cardiac development. If glucose is a major source of energy for the early fetal heart, its role extends beyond that of energy substrate. Glucose is involved in the pentose phosphate pathway and nucleotide biosynthesis. It was recently shown that glucose dose-dependently inhibits cardiac maturation and favors cardiomyocyte proliferation in human embryonic stem cell-derived cardiomyocytes (Nakano et al., 2017) through nucleotide biosynthesis (Nakano et al., 2017), strongly suggesting that glycolytic metabolism contributes to cardiac growth by hyperplasia *in utero*. Interestingly, glucose uptake is drastically reduced during the late gestational and early postnatal stages creating an intracellular glucose deprivation during natural *in vivo* development (Nakano et al., 2017). The pseudo-hypoxia of the fetal heart plays a key role, through the action of the hypoxia inducible factor (HIF1) in particular, which participates in the regulation of energy metabolism (Breckenridge et al., 2013; Hollinshead and Tennant, 2016), cell proliferation (Guimaraes-Camboa et al., 2015) and controls many maturation processes such as vasculogenesis and angiogenesis (Patterson and Zhang, 2010). It was shown recently in mice that at mid-gestation, the von Hippel-Lindau ubiquitin ligase (VHL)/HIF1 complex controls the metabolic shift from glycolysis to oxidative metabolism of the compact myocardium thus participating to the cardiac maturation (Menendez-Montes et al., 2016). The link between glucose availability and utilization and cardiac maturation seems to be the basis of perinatal cardiomyopathy associated with diabetic pregnancy (Amatayakul et al., 1970; Smoak, 2002; Corrigan et al., 2009).

In the late phase of cardiac fetal development, circulating lactate is responsible for the majority of cardiac oxygen consumption (Fisher et al., 1980; Werner and Sicard, 1987; Bartelds et al., 2000), while glucose and fatty acids oxidation contribute relatively less (Warshaw and Terry, 1970; Werner et al., 1989). In humans, it has also been shown that the fetal period is marked by a gradual increase in expression of genes involved in cardiac fatty acid metabolism (Iruretagoyena et al., 2014). The late fetal phase would be the scene of the first metabolic changes which would prepare the transition to a metabolism less dependent on glucose, a metabolism favorable to the maturation/differentiation of cardiomyocytes. What triggers these phenomena remains to be established but it is clear that metabolism plays a key role in cardiac growth and maturation.

## The Postnatal Transition to a New Energy State

The postnatal transition of the heart is characterized by an increase in the contractile demand due to the rapid growth, the increase in activity of the newborn, and the relative diminution of the cardiac mass. This results in an increase in energy demand that can only be provided by mitochondrial ATP production and lipid oxidation.

The metabolic transition occurs quickly and is triggered at least in part by birth which leads to profound changes in the blood concentration of substrates. Whereas the prenatal period is marked by a significant intake of lactate by the placenta, the first meals of the newborn are enriched in lipids from maternal milk (Girard et al., 1992), thereby leading almost immediately to the reversal of the lactate/fatty acid ratio in the blood (Lopaschuk and Jaswal, 2010). The high lipid content of the colostrum (Bitman et al., 1983) accentuates this phenomenon, which plays a major role in the transition of the cardiomyocyte metabolism from carbohydrate to lipid metabolism. The great upheavals (circulatory, respiratory and nutritive) of the first moments of life are the starting point of cellular maturation that ensures the establishment of a highly oxidative metabolism necessary for the development of the postnatal heart (Chung et al., 2007; de Carvalho et al., 2017).

Postnatal development is actually marked by an increase in the cardiac mitochondrial mass and the expression of mitochondrial proteins including those controlling the fusion/fission mechanisms (Hallman, 1971; Glatz and Veerkamp, 1982; Taha and Lopaschuk, 2007; Piquereau et al., 2010; Martin et al., 2014). Note that deletion of both mitofusin 1 and 2, the proteins involved in mitochondrial fusion, postnatally leads to ultrastructural disorganization and heart failure (Papanicolaou et al., 2012). In general, in animals as well as in humans, many genes involved in mitochondrial biogenesis have increased expression during the first weeks of life (Pohjoismaki et al., 2012, 2013). This is combined with the strengthening of the processes responsible for the degradation of fatty acids, in particular due to an increase at birth of the expression of the peroxisome proliferator-activated receptor α (PPARα) (Barger and Kelly, 2000; Lehman et al., 2000), which is activated by fatty acids and increases expression of enzymes and transporters required for fatty acid oxidation (for review, see Warren et al., 2017), the lipids becoming rapidly (in just a few days in the rodent and rabbit) the main energy source of the cardiomyocyte (Lopaschuk et al., 1992; Fukushima et al., 2016).

Following the increase in mitochondrial mass, the postnatal heart also strengthens the mechanisms protecting the cells against possible oxidative damage (Das et al., 1987; Bodi et al., 2017) since mitochondria are the main source of reactive oxygen species (ROS) (Taverne et al., 2013). While the latter play an important role in maturation processes (Murray et al., 2014), they can also cause intracellular damages affecting cardiomyocyte functions (Tsutsui et al., 2011; Hafstad et al., 2013), thereby leading the cardiac muscle cell to develop defense mechanisms.

During the cardiac fetal-to-adult transition, the remarkable increase in mitochondrial biogenesis and maturation, as well as the dramatic shift in substrate utilization are controlled by nuclear receptor signaling that responds to developmental signals and postnatal physiologic conditions. A subset of cardiac-enriched nuclear receptors serves to match mitochondrial fuel preferences and capacity for ATP production with changing energy demands of the heart (Vega and Kelly, 2017). Moreover, important post-translational modifications like acetylation and succinylation of key metabolic enzymes and transcription factors play a crucial role in maturation of cardiac energy metabolism (Fukushima et al., 2016).

## Toward Intracellular Complexity and Increased Efficiency

While development is the stage of obvious metabolic maturations, it is also a period marked by profound evolutions of the intracellular structure of the cardiomyocyte. Establishment of the definitive architecture of the cardiomyocyte is a long process that takes place during all the prenatal period but also during the early phase of postnatal development. The early postnatal growth is achieved by hyperplasia and is followed by a second phase involving hypertrophy of the cardiomyocyte which occurs very rapidly (3 days after birth in the mouse heart) (Leu et al., 2001). The cardiac cell thus passes from a polygonal shape to an elongated form (Leu et al., 2001; Hirschy et al., 2006) and its intracellular organization becomes more and more complex with, in particular, a significant increase in the amount of intracellular structures like myofilaments, sarcoplasmic reticulum and mitochondria (Piquereau et al., 2010; Porter et al., 2011). Whereas the spatial organization of these entities seems rather random at birth, major rearrangements occur during the early phase of postnatal development to establish a highly organized cytoarchitecture adapted to the functions of the mature cardiomyocyte (Piquereau et al., 2010). In mouse, a marked change is the drastic decrease in the cytosolic compartment from more than 30% after birth to less than 2% in adult heart (Piquereau et al., 2010). The increase in myofilament volume as well as the development of the sarcoplasmic reticulum network and the structures involved in excitation–contraction coupling provide the cardiomyocyte with a compartmented Ca^2+^ handling system and efficient amplification of the calcium signal. In parallel, mitochondrial volume increases and mitochondria which appear as isolated ellipsoids or tubules in embryonic cardiomyocytes, reorganize into a reticular network in the adult heart (Muhlfeld et al., 2006). The result is the emergence of an organized mitochondrial network allowing the formation of energy micro-domains.

## Traveling in the Intracellular Environment

It is clear that the metabolic pathways selected during development allow optimal energy production at each moment within the cardiomyocyte and this would be useless without efficient transfers of this energy. In the complex architecture of adult cardiomyocytes, where the main energy consumers are sarcoplasmic reticulum ATPases (SERCA) and ATPases of myosin myofilaments (myosin-ATPases), the energy must indeed be transferred from the mitochondrial compartment to these two ATPases (Ventura-Clapier et al., 1998). However, this is not as easy as it seems since the high density of myofilaments and mitochondria and the dense structure of the mature cardiomyocyte largely limit diffusion phenomena (Saks et al., 2008).

Early on, Bessman suggested that CK may play the role of a “shuttle” carrying energy within the striated muscle cell (Bessman and Geiger, 1981). This enzyme, responsible for the reversible transfer of the high energy bound of ATP to creatine (Cr) (ATP + Cr ↔ ADP + PCr) is at the heart of a system of energy transfer that is based on a complex organization of the different isoforms of CK (**Figure 2**). Cytosolic CK [predominantly represented by the MM-CK isoenzyme in the heart (Wallimann et al., 1992)] is either free in the cytoplasm or bound near ATPases of myofilaments and sarcoplasmic reticulum (Wallimann and Eppenberger, 1985; Rossi et al., 1990), whereas mitochondrial CK (mi-CK) is located in the mitochondrial intermembrane space near the adenine nucleotide translocase (ANT) (for reviews, see Saks et al., 1977; Wallimann et al., 1992; Schnyder et al., 1994). Insofar as ANT ensures the importation of ADP into the mitochondrial matrix and the export of the newly synthesized ATP to the intermembrane space (Saks et al., 1994), it supplies the mi-CK with ATP which can then produce PCr in the vicinity of the mitochondria. In the adult heart, nearly 90% of energy leaves mitochondria in the form of PCr, not ATP. The high-energy phosphate moiety of PCr is transferred, via cytosolic CK and the bound MM-CK, near ATPases where it can then be used to rephosphorylate the locally produced ADP to adjust the ATP/ADP ratio. Interestingly, this compartmentation of CK isoenzymes close to ATPases increases the local ATP/ADP ratio which thermodynamically and kinetically favors ATPases activity by relieving inhibition by-products (low inhibition constant for ADP) and providing high concentration of substrate (ATP). Close to the ANT in the energy production compartment, enzymatic coupling between mi-CK and translocase produces a high ADP/ATP ratio favorable to stimulate oxidative phosphorylation. This mechanism is the main pathway by which oxygen consumption adjusts to cardiac work (Saks et al., 2006a). While this shuttle is very efficient in transporting energy to the consumption sites, it also allows energy consumption information to be brought to the mitochondria where an accumulation of Cr stimulates the production of ATP, thereby ensuring perfect coordination of energy production and consumption (Saks et al., 1991; Ventura-Clapier et al., 1998). Other shuttles based on compartmentalized energy transfer systems are also present in the cardiomyocyte like the adenylate kinase system, or the nucleotide diphosphokinase (NDPK) system (Dzeja and Terzic, 2003).

Although highly effective, the phosphotransfer shuttles are not the only systems that optimize energy transfer within the cardiomyocyte. In the early 2000s, it was shown in rodents that the close arrangement of cellular organelles (mitochondria, sarcoplasmic reticulum, and myofilaments) allows the emergence of energetic micro-domains where the transfer of nucleotides is more efficient than the mere diffusion of this compounds through the cytoplasm (Kaasik et al., 2001; Wilding et al., 2006) (**Figure 2**). This mechanism, called direct adenine nucleotide channeling (DANC), also maintains a favorable ATP/ADP ratio in the vicinity of ATPases. This transfer system, which makes possible to overcome ADP diffusion hindering (Jacobus, 1985), plays a significant role in the energetic supply of ATPases since it displays a yield comparable to the CK system (Kaasik et al., 2001). These two energy transfer systems constantly compete with each other. In physiological conditions, the CK system is predominant and provides about two-thirds of the energy transfer when the DANC only participates for one-third (Kaasik et al., 2001). These proportions are not frozen since CK shuttle and DANC can compensate one another when required by the conditions (Ventura-Clapier et al., 2004; Tylkova, 2009).

## Establishment of Energy Transfer Systems During Postnatal Development

**FIGURE 3:**
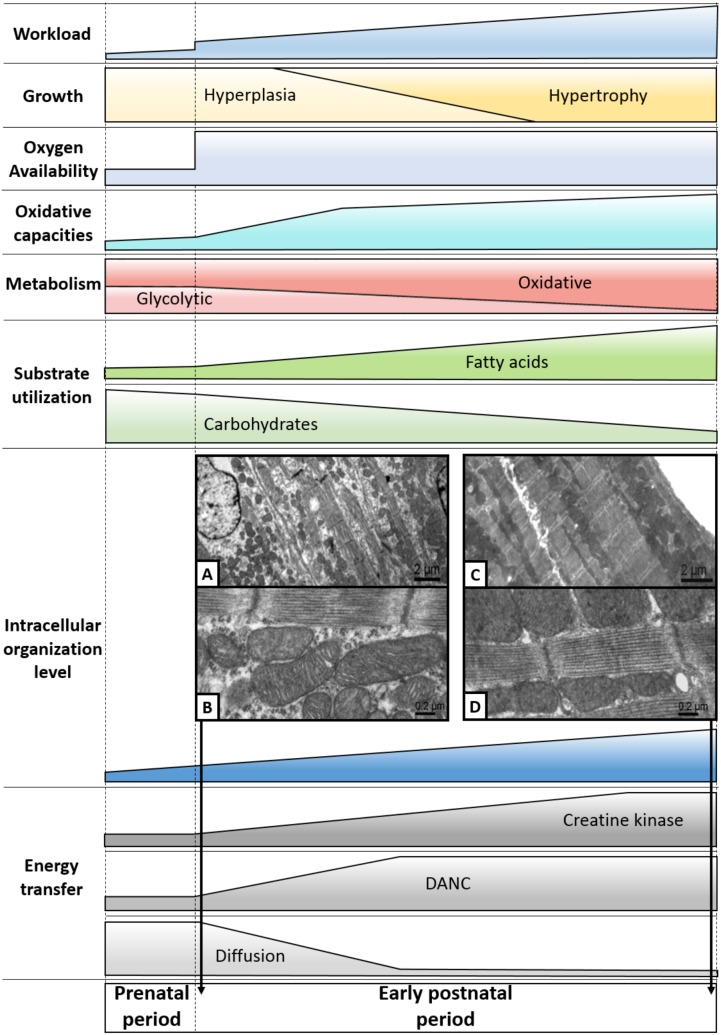
Highlights of the maturation of cardiac energy metabolism. During the cardiac perinatal development, the heart adapts to the increased workload by increasing its size by hyperplasia and then by hypertrophy. Over this period, the heart undergoes profound metabolic upheavals, notably marked by a significant increase in its oxidative capacities. This leads to the progressive establishment of a metabolism that is overwhelmingly oxidative and based on the use of fatty acids. Then, efficient energy transfer systems emerge, correlating with the appearance of an intracellular architecture displaying an increasingly complex level of organization. Pictures **A** and **B** are electron micrographs of 3-day-old cardiomyocytes of papillary muscle while pictures **C** and **D** are electron micrographs of 63-day-old cardiomyocytes of papillary muscle (adapted from Piquereau et al., 2010 with permission).

These efficient energy transfer systems do not seem to operate in the fetal heart. The production of energy mainly dependent on glycolysis and the organization of glycolytic enzymes in supramolecular complexes in the vicinity of ATPases allows an effective control of the local ATP/ADP ratio (Brooks and Storey, 1991; Ventura-Clapier et al., 2011). Therefore, this does not require a particular transfer system. The relatively loose cellular architecture of the fetal cardiomyocyte (Hirschy et al., 2006; Lozyk et al., 2006) is also much more favorable for the diffusion of energy molecules that does not require a real transfer system to reach the consumption and production sites (**Figure 3**). Moreover, the energy fluxes are much lower and can accommodate the low diffusion rate. The fetal heart can thus operate without these highly organized transfer mechanisms (Hoerter et al., 1991, 1994; Tiivel et al., 2000).

In view of the complex mechanisms responsible for energy supply in adult cardiomyocytes, it is clear that a maturation phase ensures the establishment of the aforementioned energy transfer systems. While the very low CK activity of the fetal heart prohibits any transfer by the CK shuttle (Hoerter et al., 1991, 1994; Fischer et al., 2010), the important increase in CK activity around birth, associated with an increase in the cellular content of creatine allows a progressive implementation of this phosphotransfer system (Hoerter et al., 1991; Fischer et al., 2010; Anmann et al., 2014). Progressively with the increase in CK activity, the complexification of the intracellular structure and the increase in energy demand, the CK system becomes able to support the work of ATPases and to ensure an effective positive feedback on energy production (Hoerter et al., 1991; Piquereau et al., 2010; Anmann et al., 2014) (**Figure 3**). During the early postnatal period, the mitochondrial permeability to ADP decreases in parallel with the increased efficiency of mi-CK, while MM-CK is progressively compartmentalized in myofilaments, allowing mitochondrial respiration to be under the control of the CK shuttle (Hoerter et al., 1991). Changes in mitochondrial sensitivity to ADP during postnatal development seems to be linked to cytoskeletal rearrangement but further studies are needed to determine which cytoskeletal proteins are needed (Anmann et al., 2014). Studies on different species seem to show that effectiveness of the CK shuttle would depend on the general state of maturity of the cardiac cell rather than the physiological changes induced by birth. It has been shown that the crucial phase of CK compartmentation takes place in the first 3 weeks of postnatal life in mice or rabbit while these systems would be able to support the ATPase activity of myofilaments before birth in the guinea pig, a species whose maturation state is more advanced at birth (Hoerter et al., 1994).

As the DANC is dependent on the architecture of the cardiomyocyte which ensures a precise arrangement of the organelles (Wilding et al., 2006; Piquereau et al., 2010, 2012), it is obvious that this system of energy transfer only occurs during the architectural maturation of the cardiomyocyte. The transition from the loose spatial organization of these entities at birth to the complex cytoarchitecture established during the early phase of postnatal development results in the emergence of an organized mitochondrial network allowing the formation of energy micro-domains (Piquereau et al., 2010). In mouse, this reorganization takes place in the first week of postnatal life, a pivotal period for the establishment of an effective DANC (Piquereau et al., 2010). Interestingly, in mice the DANC and the CK shuttle mature before the hypertrophic phase of the neonatal heart around 11 days after birth (Alkass et al., 2015). This seems to suggest that the hyperplasic phase of the heart growth corresponds to the period when the cardiac cell acquires its adult characteristics before beginning the hypertrophic phase of growth that would allow a mature heart to increase its mass as the body grows. The link between cytoarchitecture and energy metabolism is highlighted by the architectural remodeling induced by CK deficiency. In the heart of CK null mice, mitochondria are reorganized within myofilaments thus decreasing diffusion distances and increasing DANC efficiency, showing that subcellular organization is sensitive to energy deficiency (Kaasik et al., 2001).

## Conclusion

The study of the metabolism of the cardiac cell during the development shows how the cellular energy is reactive to the evolution of the organ. The constant increase in cardiac workload leads to an increase in the contractile capacity of the cardiomyocyte which adapts on the one hand by moving toward a more efficient energy source (lipids) and on the other hand by implementing highly specialized energy transfer systems due to the density of emerging cytoarchitecture (**Figure 3**). Interestingly, metabolic maturation precedes the maturation of excitation–contraction coupling as a prerequisite for efficient energy yield. There is much evidence that energy metabolism plays a major role to drive cardiac cell maturation and cytoarchitecture but their exact interaction and triggers remain to be established. It seems that the period of hyperplasia of cardiac growth is the scene of the acquisition of adult physiological characteristics of the cardiomyocyte, whether metabolically or morphologically. Synchronization of the transition between hyperplasic and hypertrophic growths with the establishment of cellular architecture and the emergence of oxidative metabolism is not surprising. While it is conceivable that the pseudo-organized neonatal cardiomyocyte can divide in order to increase cardiac muscle mass at the beginning of postnatal life, this process seems to be unlikely for a cell having already acquired a cellular architecture presenting the complexity of the adult cardiomyocyte. Densification of the internal structures of the cardiomyocyte would thus be a major component of the transition to strict hypertrophic cardiac growth (Li et al., 1997a,b). The latter would only increase the density of myofilaments an SR and the amount of mitochondria, without major metabolic changes.

## Author Contributions

JP wrote the first version of the review. JP and RV-C wrote the final version.

## Conflict of Interest Statement

The authors declare that the research was conducted in the absence of any commercial or financial relationships that could be construed as a potential conflict of interest.
